# High early fluid and sodium intake as risk factors for acute kidney injury in very-low-birthweight infants

**DOI:** 10.1007/s00467-025-07049-w

**Published:** 2025-11-20

**Authors:** Pauliina M. Mäkelä, Lotta Immeli, Markus Leskinen, Reijo Sund, Timo Jahnukainen, Sture Andersson, Päivi Luukkainen

**Affiliations:** 1 Pediatric Research Center, New Children’s Hospital, Helsinki University Hospital, and University of Helsinki, Helsinki, Finland; 2 Department of Pediatric Nephrology and Transplantation, New Children’s Hospital, Helsinki University Hospital, Stenbäckinkatu 9, P.O. Box 347, FIN-00029 HUS Helsinki, Finland; 3 School of Medicine, Institute of Clinical Medicine, University of Eastern Finland, Kuopio, Finland

**Keywords:** Infant, Premature, Infant, Very low birth weight, Kidney injury, Acute, Water–Electrolyte balance, Weight loss

## Abstract

**Background:**

Acute kidney injury (AKI) is common in very-low-birthweight (VLBW) infants. Both fluid overload and dehydration can lead to AKI. Our aim was to examine the associations between early fluid and sodium intake and AKI.

**Methods:**

This retrospective cohort study comprised 421 VLBW infants born at < 32 weeks. Detailed data on fluid management during the first 24 h of life, diuresis and weight changes during the first postnatal week and plasma creatinine measurements during the first 2 postnatal weeks were acquired from an electronic patient information system. AKI was defined according to the KDIGO definition modified for neonates.

**Results:**

The incidence of AKI was 8.6%, and on average, it was diagnosed on the fifth postnatal day. Higher total fluid intake (mL/kg/24 h) during the first day of life was associated with an increased risk of AKI (OR, 1.015; 95% CI, 1.005–1.025; *p* < 0.01), analysis adjusted for gestational age and being small-for-gestational age. The highest fluid intake quartile had a 5.6-fold risk of developing AKI when compared with the lowest quartile (*p* = 0.01). A higher total sodium intake (mmol/kg/24 h) was associated with an increased risk of AKI (OR, 1.12; 95% CI, 1.03–1.21; *p* < 0.01). Among infants with AKI, a substantial proportion of early fluid and sodium intake (median 27% and 59% of total intake, respectively) originated from volume expanders. Infants diagnosed with AKI exhibited an average weight gain of + 2.8% by the second day of life.

**Conclusions:**

As part of efforts to minimize the risk of AKI, avoiding excessive fluid and sodium administration during the first postnatal hours may be beneficial.

**Graphical abstract:**

**Figure:**
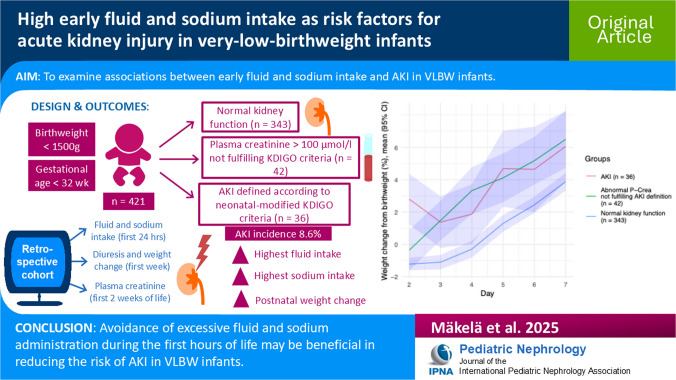
A higher resolution version of the Graphical abstract is available as Supplementary information

**Supplementary Information:**

The online version contains supplementary material available at 10.1007/s00467-025-07049-w.

## Introduction

Acute kidney injury (AKI) is a common finding in the neonatal intensive care unit (NICU), and it has been shown to be an independent risk factor for increased length of hospital stay and mortality in neonates [1]. The Assessment of Worldwide Acute Kidney Injury Epidemiology in Neonates (AWAKEN) study, a multicenter, multinational retrospective cohort study conducted in 2014, reported an incidence of AKI of 30% among critically ill neonates [2]. Very low birthweight (VLBW, birthweight < 1500 g) infants with immature kidney function are at particular risk of developing AKI, the risk further increased by the frequent use of nephrotoxic medications in this population [3, 4]. It has been shown that lower gestational age is associated with a higher risk of AKI in VLBW infants [3]. The reported incidences of AKI in VLBW infants vary between 18 and 40% [1, 5, 6], the variation in incidences is partly explained by the nonuniform definitions of AKI used.

In preterm infants, insensible water loss is high, and the immature kidneys have limited capacity to excrete waste products and regulate fluid balance [7, 8]. Therefore, VLBW infants are prone to both fluid overload and dehydration. Optimal fluid balance is an important factor in preventing kidney injury, since both fluid overload and dehydration can lead to AKI. Dehydration and hypotension reduce kidney perfusion and may provoke AKI through ischemic injury [9]. On the other hand, fluid overload has been identified as an independent risk factor for AKI in critically ill patients both in pediatric and adult populations [10, 11].

In VLBW infants, data are scarce regarding the impact of early fluid management on the risk of AKI. For the past two decades, our unit has been using an electronic patient information system that systematically collects information on, for example, fluid and electrolyte intake, blood pressures and diuresis. Our aim was to describe the incidence of neonatal AKI in a cohort of VLBW infants and to examine the associations between early fluid and sodium intake and AKI. We hypothesized that the incidence of AKI would be higher among infants receiving either low or high fluid volumes, compared to those with a more moderate intake.

## Patients and methods

### Study design and patients

We conducted this retrospective cohort study as part of the Big Data – Tiny Infants research project. All infants with a birthweight below 1500 g admitted to the tertiary care NICU of the Helsinki University Children’s Hospital between January 1, 2005, and December 31, 2013 were identified and included in the cohort, when meeting the following criteria: gestational age (GA) below 32 weeks, no gastrointestinal or other major malformations or chromosomal abnormalities, admission to the NICU during the first 24 h of life and spending at least one full 24-h period in the NICU, and having their plasma creatinine level measured at least twice during the first 2 weeks of life.

### Data collection

All plasma creatinine measurements from birth until postnatal day 14, as well as data on diuresis and weight during the first postnatal week, were retrospectively acquired from the NICU’s electronic patient information system, Centricity Critical Care Clinisoft (GE Healthcare, Chicago, IL, USA). During the study period, creatinine was measured from lithium-heparin plasma samples using an enzymatic assay traceable to the reference method of isotope dilution mass spectrometry (IDMS). Changes in plasma creatinine levels were assumed to be like those in serum. We identified infants with AKI using modified, neonatal Kidney Disease: Improving Global Outcomes (KDIGO) criteria [12–14], as adapted in the AWAKEN study [2]. AKI was defined as any of the following:

1. increase in serum creatinine by ≥ 26.5 µmol/L (≥ 0.3 mg/dL) within 48 h; or
2. increase in serum creatinine to ≥ 1.5 times baseline, which is known or presumed to have occurred within the prior 7 days; or
3. urine volume less than 1 mL/kg birthweight/h for at least 6 h on postnatal days 2 to 7.

As urine output data were available only in 24-h increments, we used a urine output definition of less than 1 mL/kg/h for 24 h. All the weight measurements during postnatal days 2 to 7 were acquired and the weight change from birthweight was calculated as (weight measured on a given day − birthweight)/birthweight. To evaluate the hemodynamic status on the first day of life, we also collected the invasive mean arterial pressure (MAP) data generated by measuring devices that automatically record and store the arterially measured blood pressure as 2-min averages. This blood pressure data contained 274,090 measurements for the cohort’s infants during the first day of life. Since there were artifacts due to arterial blood sampling and operational problems with the arterial cannula, 15,528 recordings were removed as outliers.

To examine the early fluid and sodium intake, all parenteral and enteral fluids given to each infant during the first postnatal day (i.e., the first 24 h of life) were acquired from the electronic patient information system containing detailed, timestamped records of all fluids and medications given. We also retrieved plasma sodium measurements during the first 48 h of life to examine the plasma sodium concentrations before and after the fluids administered during the first postnatal day. According to our NICU’s clinical practice, the water content of breast milk and formula was estimated to be 80%. Sodium intake was calculated from manufacturer information and published electrolyte content [15]. For each infant, we calculated the cumulative fluid and sodium intake during the first 24 h of life, and we also examined parenteral and enteral intake, as well as fluid and sodium deriving from volume expanders, separately. The fluid and sodium intakes were adjusted by dividing the amount by birthweight and are thus given as mL/kg birthweight or mmol/kg birthweight. In addition, for supplementary analyses, data on fluid and sodium intake for the entire first week of life were collected, and cumulative intakes (mL/kg/wk and mmol/kg/wk) were calculated for those with complete data available (*n* = 359).

The nutritional practices in our unit followed the European guidelines on parenteral and enteral nutrition [16–18]. Ringer’s solution, fresh frozen plasma, 4–5% albumin solution and sterile water were used as volume expanders, if necessary. Data on inotropes and nephrotoxic medications during the first 24 h of life were collected. The primary inotrope used in our unit was dopamine; dobutamine, noradrenaline and adrenaline were used as vasopressors to a lesser extent. Nephrotoxic medications included non-steroidal anti-inflammatory agents (NSAIDs) for patent ductus arteriosus closure (indomethacin and ibuprofen), antibiotics (aminoglycosides and vancomycin) and antiviral agents (acyclovir).

Clinical characteristics and diagnoses, except for sepsis, were acquired from the Finnish Medical Birth Register Data on Premature Infants, a nationwide register administered by The Finnish Institute of Health and Welfare (THL). Gestational age (GA) was determined from the first day of the last menstrual period, and in 88% of the cases, it was confirmed by ultrasonography at 11 + ^0/7^–13 + ^6/7^ weeks of gestation. Small for gestational age (SGA) was defined as a birthweight Z-score of <  − 2 standard deviations on the Finnish growth charts [19]. Patent ductus arteriosus (PDA) was diagnosed with ultrasound and included in the analyses only if surgical intervention was needed. Intraventricular hemorrhages (IVH) were graded according to Papile classification [20], and grade III to IV hemorrhages were included in the analyses. Bronchopulmonary dysplasia (BPD) was defined as a need for supplemental oxygen at 36 weeks’ postmenstrual age [21]. Necrotizing enterocolitis (NEC) was defined according to modified Bell’s staging criteria as Bell stage II or higher. Data on sepsis were retrieved from the patient information system and required a positive blood culture in combination with a plasma C-reactive protein level > 10 mg/L on the day the positive blood culture sample was drawn, or on the two succeeding days [22].

### Statistics

Descriptive data are presented as numbers and percentages, medians and interquartile ranges (IQR), or means and standard deviation (SD), where appropriate. The chi-square test and Fisher’s exact test were applied for categorical data. Due to skewed distributions, fluid and sodium intake data were analyzed with the non-parametric Kruskal–Wallis and Mann–Whitney *U* tests. The p values in these analyses were corrected for multiple comparisons with Bonferroni correction. The normally distributed postnatal weight change data were analyzed with repeated measures ANOVA, and Tukey’s HSD was applied as a post hoc test. Associations between fluid and sodium intake and the risk of AKI were calculated using logistic regression. Separate models were fitted for fluid and sodium, and the models were adjusted for GA, SGA status, and average MAP during the first 12 h of life. The results of logistic regression models are presented as adjusted odds ratios (OR) and 95% confidence intervals (CI). A corrected *p*-value of < 0.05 was considered statistically significant. Statistical analyses were conducted using R4.3.2 statistical software (R Foundation for Statistical Computing).

### Ethical considerations

The Ethics Committee of the Helsinki University Hospital approved the study protocol with approval number 115/13/03/00/2014. Informed consent was waived, as all the data were pseudonymized.

## Results

**Fig. 1:**
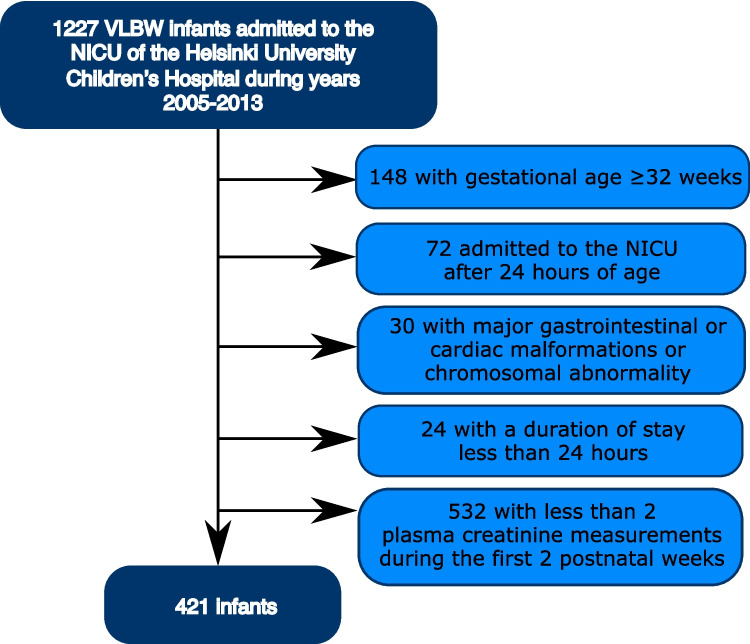
Flow chart of the patients

Between 2005 and 2013, a total of 1227 VLBW infants were admitted to the NICU of the Helsinki University Children’s Hospital. Of these infants, 421 met the inclusion criteria of our study (Fig. 1). Six infants in the cohort were diagnosed with congenital urinary tract anomalies; the diagnoses included unilateral kidney dysplasia, unilateral kidney agenesis, hydronephrosis, hypospadias and chordee. However, exclusion was not deemed necessary, since these conditions were not expected to significantly influence early fluid management or the risk of neonatal AKI in these cases. During the first 2 weeks of life, all infants had their plasma creatinine levels measured at least twice, with the median number of measurements being 3 per infant (IQR 2–4, range 2–16).

### Incidence of acute kidney injury

**Fig. 2:**
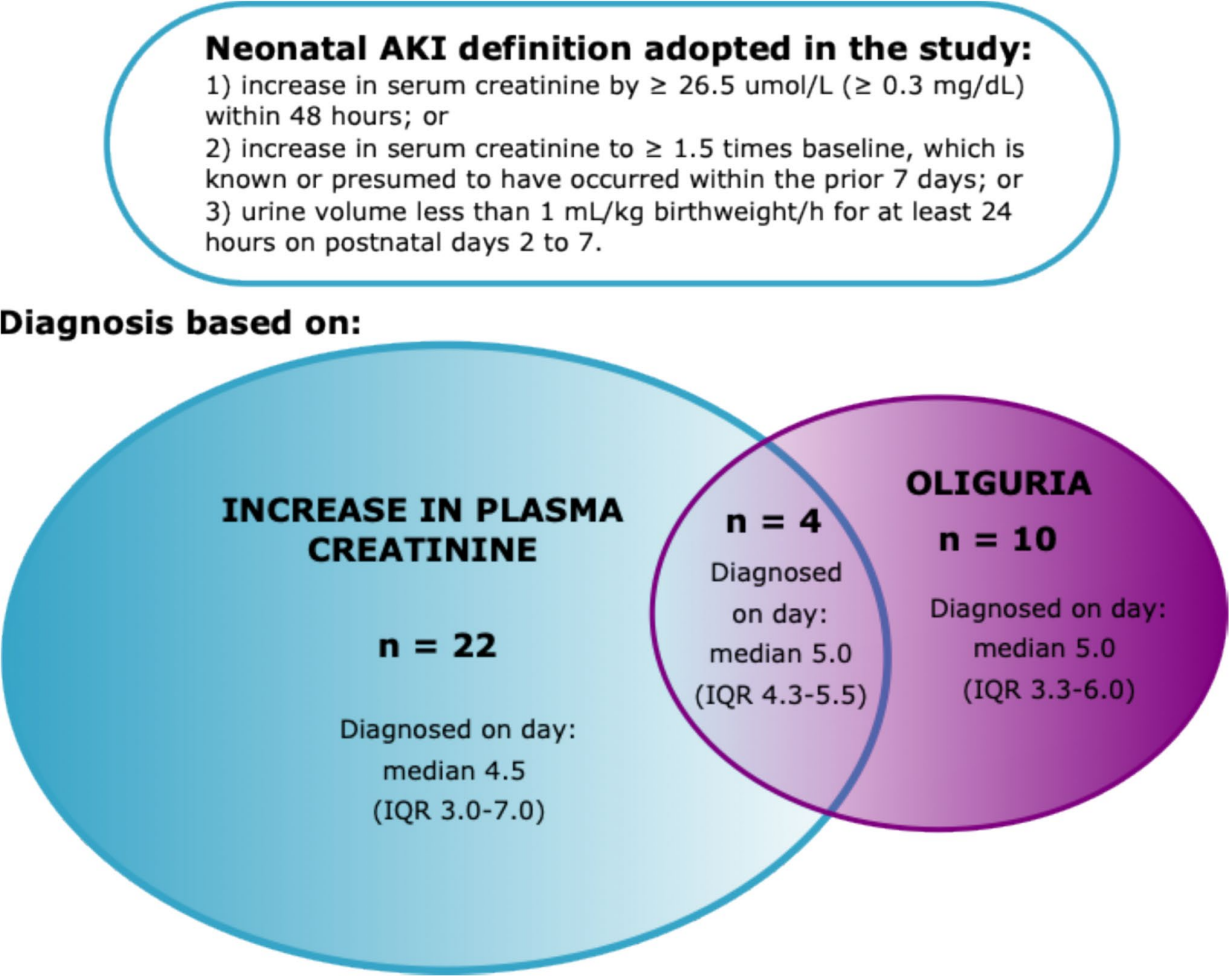
Neonatal AKI definition and distribution of diagnoses by fulfilled criteria

Of the cohort’s 421 infants, 36 (8.6%) infants were diagnosed with AKI according to the modified KDIGO definition [2]. On average, AKI was diagnosed on the fifth postnatal day (median 5, IQR 3–7, range 1–10). Of the AKI diagnoses, 22 were based solely on the increase of plasma creatinine levels and 10 solely on oliguria (diuresis less than 1 mL/kg/h for at least one 24-h period during postnatal days 2 to 7), with 4 patients meeting both the creatinine increase and the oliguria criteria (Fig. 2). We also identified 42 infants (10.0% of the cohort) with markedly elevated plasma creatinine (P-Crea) levels above 100 µmol/L at least once during postnatal days 3 to 14, without meeting the AKI criteria. When identifying the infants with markedly elevated P-Crea levels, we left out the creatinine measurements of the first two postnatal days, since those might reflect the mother’s kidney values. In the analyses, these infants with markedly elevated P-Crea values were examined as a separate group, since their kidney function could not be regarded as completely normal. Thus, for the analyses, the infants were divided into three groups: infants with AKI (*n* = 36), infants with abnormal P-Crea not fulfilling AKI definition (*n* = 42) and infants with normal kidney function (*n* = 343). When identifying the infants with AKI, all creatinine measurements from birth until postnatal day 14 were included, since while baseline levels may be influenced by maternal creatinine, any subsequent increase reflects the kidney function of the neonate. Clinical characteristics of the infants are shown in Table 1. There were no significant differences in the three groups regarding gender, gestational age, birthweight, SGA status, duration of mechanical ventilation, or the incidence of sepsis during the first postnatal week or the incidences of BPD, PDA, NEC, and IVH.

**Table 1:** Clinical characteristics

	AKI (*n*= 36)	Abnormal P–Crea not fulfilling AKI definition (*n* = 42)	Normal kidney function (*n* = 343)	*p*-value
Male, *n* (%)	20 (56)	26 (62)	188 (55)	0.68*
Gestational age (wk), median (min–max)	27.1 (23.3–31.3)	26.8 (23.0–30.9)	27.3 (23.0–31.9)	0.26**
Birthweight (g), median (min–max)	930 (520–1455)	965 (450–1480)	940 (400–1490)	0.71**
Small for gestational age, *n* (%)	5 (14)	6 (14)	69 (20)	0.47*
Multiple births, *n* (%)	13 (36)	15 (36)	80 (23)	0.07*
Cesarean section, *n* (%)	22 (61)	27 (64)	238 (69)	0.51*
Arterial pH at birth (median, IQR)ª	7.30 (7.26–7.37)	7.30 (7.24–7.32)	7.30 (7.25–7.35)	0.27**
Apgar score < 7 at 5 min, *n* (%)	21 (58)	22 (52)	159 (46)	0.70***
Maternal diabetes, *n* (%)	1 (3)	4 (10)	31 (9)	0.31***
Maternal pre-eclampsia, *n* (%)	4 (11)	7 (17)	87 (25)	0.19***
Antenatal corticosteroid given, *n* (%)	35 (97)	40 (95)	325 (95)	0.06***
Surfactant given, *n* (%)	36 (100)	41 (98)	306 (89)	0.02***
Duration of mechanical ventilation (days), median (IQR)	9.0 (4.0–16.5)	11.0 (4.0–27.8)	8.0 (3.0–28.0)	0.77**
Inotrope use within the first 24 h, *n* (%)	31 (86)	31 (74)	209 (61)	0.004*
Bronchopulmonary dysplasia, *n* (%)	8 (22)	16 (38)	125 (36)	0.34***
Patent ductus arteriosus, surgically treated, *n* (%)	12 (33)	14 (33)	103 (30)	0.88*
Necrotizing enterocolitis, *n* (%)	2 (6)	4 (10)	25 (7)	0.77***
Intraventricular hemorrhage, *n* (%)	7 (19)	6 (14)	44 (13)	0.48***
Culture positive sepsis during the first week of life, *n* (%)	0 (0)	1 (2)	11 (3)	0.85***
Died during the first 2 weeks of life, *n* (%)	6 (17)	3 (7)	15 (4)	0.01***
Died before age 28 days, *n* (%)	6 (17)	4 (10)	19 (6)	0.03***

Statistical tests: *Chi-squared test, **Kruskal–Wallis test, ***Fisher’s test. *AKI* acute kidney injury, *P-Crea* plasma creatinine, *wk* week, *g* gram, *IQR* interquartile range. ^a^ Missing data (*n* = 68: 5 from the AKI group, 10 from the abnormal P-Crea group, and 53 from the normal kidney function group)

During the first 24 h of life, there was an average of 623 arterial blood pressure recordings for each infant. The lowest MAP during the first day of life with a median of 22 mmHg occurred in the AKI group at the median age of 4.7 h, while the corresponding values were 23 mmHg at the age of 8.3 h in the abnormal P-Crea not fulfilling the AKI definition group and 25 mmHg at the age of 4.3 h in the normal kidney function group. In comparison with the normal kidney function group, the MAP nadir was significantly lower in the AKI group (*p* < 0.01) and in the abnormal P-Crea group (*p* = 0.03).

### Early fluid intake and the risk of AKI

Table 2 presents the total, parenteral and enteral fluid intakes, as well as fluid from volume expanders during the first 24 h of life in the three study groups. During the first 24 h after birth, total fluid intake was significantly higher in the AKI group compared with the infants with normal kidney function (*p* = 0.03). Total fluid intake in infants with abnormal P-Crea not fulfilling the AKI definition did not differ statistically significantly from the other two groups. The proportion of fluid intake from volume expanders was highest in the AKI group, constituting a median of 27% of total intake in the first day of life. The incidence of inotrope use was highest in the AKI group (Table 1).

**Table 2:** Fluid and sodium intake during the first 24 h of life

Intakes during the first 24 h of life: median (IQR)	AKI (*n* = 36)	Abnormal P-Crea not fulfilling AKI definition (*n* = 42)	Normal kidney function (*n* = 343)	*p*-value (Kruskal–Wallis)	Pairwise comparison (Mann–Whitney *U* test), adjusted *p*-values
Total fluid intake (mL/kg/24 h)	138 (117–156)	133 (108–155)	122 (106–145)	0.02	0.03 (AKI vs. normal kidney function)
Parenteral fluid intake (mL/kg/24 h)	131 (109–149)	122 (100–140)	111 (95–133)	0.003	0.004 (AKI vs. normal kidney function)
Enteral fluid intake (mL/kg/24 h)	7 (5–11)	10 (7–15)	11 (8–15)	0.004	0.003 (AKI vs. normal kidney function)
Volume expanders (mL/kg/24 h)	39 (28–64)	28 (22–48)	29 (18–42)	0.01	0.01 (AKI vs. normal kidney function)
Volume expanders of total fluid intake (%)	27 (23–40)	23 (17–33)	24 (17–31)	0.03	0.03 (AKI vs. normal kidney function)
Total sodium intake (mmol/kg/24 h)	7.7 (5.2–10.9)	6.4 (5.4–9.4)	6.5 (4.6–8.3)	0.04	0.048 (AKI vs. normal kidney function)
Sodium from volume expanders (mmol/kg/24 h)	4.6 (2.9–8.3)	3.5 (2.7–5.1)	3.5 (2.0–5.1)	0.03	0.03 (AKI vs. normal kidney function)
Volume expander derived sodium from total (%)	59 (47–73)	56 (46–66)	55 (44–66)	NS (0.29)	NS

Higher total fluid intake during the first day of life was associated with an increased risk of AKI (OR, 1.015; 95% CI, 1.005–1.025; *p* < 0.01), with each 1 mL/kg increase in fluid intake increasing the risk of AKI by 1.5%, after adjusting for GA and SGA status. When examining total fluid intake in quartiles, logistic regression showed that the highest fluid intake quartile had a 5.6-fold risk of developing AKI (*p* = 0.01) when compared with the lowest quartile; this analysis also adjusted for GA and SGA status.

We also examined a logistic regression model with AKI as a dependent variable and total fluid intake, GA, SGA status and the average MAP during the first 12 h of life as independent variables. In this model, higher total fluid intake remained significantly associated with increased risk of AKI (OR, 1.014; 95% CI, 1.003–1.024; *p* < 0.01, Table 3).

**Table 3:** Multivariable logistic regression model predicting the odds of acute kidney injury

		95% confidence interval		
	Odds ratio (OR)	Lower	Upper	*p*-value
Total fluid intake during the first 24 h of life (mL/kg/d)	1.014	1.003	1.024	< 0.01
Gestational age (days)	1.023	0.992	1.056	0.15
Small for gestational age (true)	0.388	0.116	1.073	0.09
Mean MAP during the first 12 h of life	0.920	0.816	1.022	0.15

### Early sodium intake and the risk of AKI

Table 2 presents the total and volume expander-derived sodium intake during the first 24 h of life in the three study groups. During the first 24 h after birth, total sodium intake was significantly higher in the AKI group compared with the infants with normal kidney function (*p* = 0.048). In logistic regression adjusted for GA and SGA status, higher total sodium intake (mmol/kg/24 h) was associated with an increased risk of AKI (OR, 1.12; 95%, CI 1.03–1.21, *p* < 0.01), with each 1 mmol/kg/24 h increase in sodium intake increasing the risk of AKI by 12%.

We also examined a logistic regression model with AKI as a dependent variable and total sodium intake, GA, SGA status and the average MAP during the first 12 h of life as independent variables. In this model, higher sodium intake was associated with an increased risk of AKI (OR, 1.12; 95% CI, 1.02–1.21; *p* < 0.01, Table 4).

**Table 4:** Multivariable logistic regression model predicting the odds of acute kidney injury

		95% confidence interval		
	Odds ratio (OR)	Lower	Upper	*p*-value
Total sodium intake during the first 24 h of life (mmol/kg/d)	1.12	1.02	1.21	< 0.01
Gestational age (days)	1.02	0.99	1.05	0.21
Small for gestational age (true)	0.41	0.12	1.14	0.12
Mean MAP during the first 12 h of life	0.91	0.81	1.02	0.12

No statistically significant differences in plasma sodium concentrations were observed between the study groups on postnatal days 1 and 2 (Supplementary Table 1). Sodium intake during the first 24 h of life did not correlate with the first plasma sodium concentration measured on either postnatal day 1 or 2 (Supplementary Figs. 1 and 2).

### Cumulative fluid and sodium intake during the first week of life and the risk of AKI

Complete data on fluid and sodium intake during the first postnatal week were available for 359 infants. For these infants, we calculated the cumulative intakes of fluid and sodium over postnatal days 1 to 7. Of these infants, 27 (7.5%) had AKI, 36 (10.0%) had abnormal plasma creatinine above 100 µmol at least once during postnatal days 3 to 14, and 296 (82.5%) had normal kidney function. There were no statistically significant differences in cumulative fluid or sodium intake between the groups (Supplementary Table  2 A). In unadjusted logistic regression analyses, no statistically significant associations were observed between cumulative fluid and sodium intake during the first postnatal week and the occurrence of AKI. The associations between cumulative fluid intake and the risk of AKI remained non-significant even when adjusting for GA and SGA status (Supplementary Table 2B). However, when adjusting for GA and SGA status, higher cumulative sodium intake during the first postnatal week was associated with an increased risk of AKI (OR, 1.034; 95% CI 1.006–1.062, *p* = 0.02) (Supplementary Table  2 C).

### Postnatal weight change

**Fig. 3:**
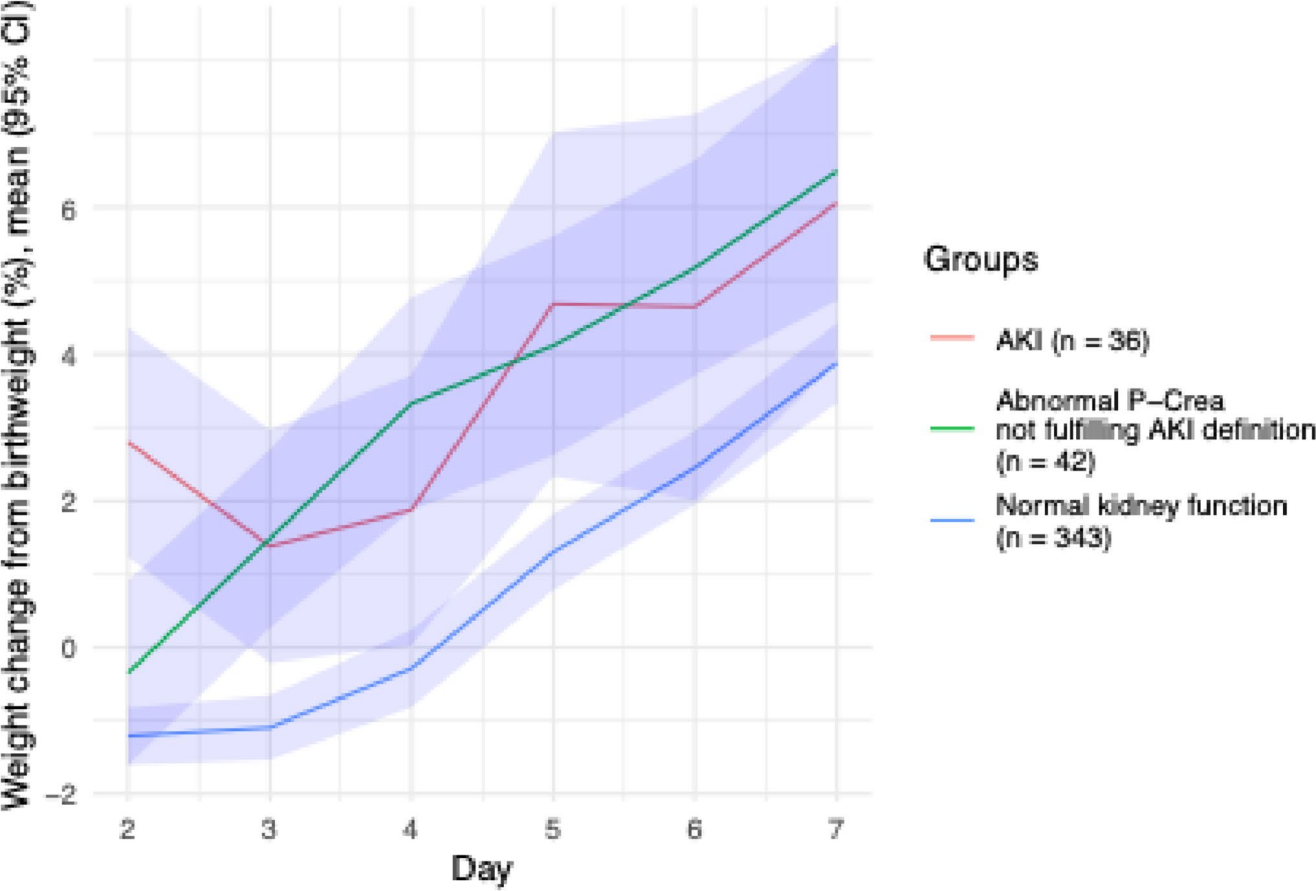
Average postnatal weight change during days 2 and 7 in the three study groups, light-blue areas indicating the 95% confidence intervals

Figure 3 shows postnatal weight changes from birthweight (%) in the three study groups during days 2 to 7. On postnatal day 2, infants with normal kidney function had an average weight loss of − 1.2% (SD 7.0%), whereas those in the AKI group had gained + 2.8% (SD 8.4%) weight from birthweight (*p* = 0.01).

### Nephrotoxic medications

During the first 24 h of life, 418 infants (99% of the cohort) received aminoglycosides as part of the antimicrobial prophylaxis. To promote the closure of PDA, 17 infants (4% of the cohort) were treated with NSAIDs during the first day of life. The highest incidences of NSAID exposure were in the abnormal P-Crea not fulfilling the AKI definition group (4/42 = 9.5%) and the AKI group (3/36 = 8.3%), whereas the incidence was significantly lower in the normal kidney function group (10/343 = 2.9%) (*p* = 0.03). All infants who received NSAIDs were also treated with aminoglycosides, increasing the nephrotoxic burden in these infants. Adding exposure to NSAIDs as an independent variable to the logistic regression models describing associations between early fluid and sodium intake and the risk of AKI, did not affect the results (Supplementary Tables 3 and 4).

## Discussion

In this cohort of 421 VLBW infants, we demonstrated that higher fluid and sodium intake during the first day of life was associated with an increased risk of AKI. The infants in the highest total fluid intake quartile had a 5.6-fold risk of developing AKI when compared with the lowest fluid intake quartile. Each 1 mmol/kg/day increase in the early sodium intake increased the risk of AKI by 12%. Among infants diagnosed with AKI, a substantial proportion of early fluid and sodium intake—median 27% and 59% of total intake, respectively—originated from volume expanders. Instead of the expected physiological weight loss, infants diagnosed with AKI during the first 2 weeks of life exhibited an average weight gain of + 2.8% by the second day of life. To our knowledge, this is the first study to specifically examine the impact of early fluid and sodium administration during the first 24 h of life on the risk of AKI in VLBW infants.

Previous studies have shown that fluid and sodium overload may predispose individuals to kidney dysfunction by promoting venous congestion and facilitating the development of interstitial edema [23, 24]. Consistent with our findings, a prospective, observational study by Askenazi et al. [25] of 58 sick near-term or term neonates (birthweight > 2000 g, GA ≥ 34 weeks) reported a higher percent weight accumulation at day 3 of life in those with AKI compared with those without AKI (median 8.2% vs. − 4%, respectively). In a retrospective analysis of 1007 premature infants (GA < 36 weeks) from the AWAKEN study, neonates with AKI were less likely to have a negative fluid balance at postnatal day 7, had consistently higher peak fluid balance during the first postnatal week and needed more frequently mechanical ventilation on day 7 [26]. Concurrent fluid overload and AKI have been suggested to significantly worsen clinical outcomes in critically ill pediatric patients [11].

As a supplementary analysis, we examined the cumulative fluid and sodium intake during the first postnatal week in infants with complete data available. We found no significant association between cumulative fluid intake during the first week of life and the risk of AKI. When adjusting for GA and SGA status, higher cumulative sodium intake was associated with an increased risk of AKI. However, the association was notably weaker compared with that observed between early sodium intake on the first day of life and the risk of AKI. The difference in these results may be partly explained by the timing of AKI diagnosis in our study, which occurred on average on the fifth day of life. Thus, the observation period for cumulative intake comprised fluid and sodium management both before and after the onset of AKI, and the diagnosis itself likely influenced subsequent fluid and sodium administration.

In our cohort of VLBW infants, the incidence of AKI was 8.6%. Compared with previous studies, the incidence in our study remained moderately low. We defined AKI according to the neonatal-modified KDIGO criteria, and in 61% of the cases the diagnosis was solely based on the serum creatinine criteria, in 28% solely on the oliguria criteria and in 11% on both criteria. In addition, up to 10% of our cohort had at least one episode of markedly elevated plasma creatinine (above 100 µmol/L) between postnatal days 3 and 14, without fulfilling the KDIGO criteria. These relatively elevated plasma creatinine concentrations observed during the early postnatal period may be attributable to a combination of immature glomerular filtration and enhanced passive tubular reabsorption of creatinine, both of which reflect the physiological immaturity of kidney function in this population. The AWAKEN study employed the same definition as in our study and reported an AKI incidence of 30% among critically ill neonates with the incidence varying by gestational age group (48% in infants born at 22 weeks to < 29 weeks, 18% in infants born at 29 to < 36 weeks and 37% in infants born at > 36 weeks) [2]. A retrospective cohort study of 455 VLBW infants by Carmody et al. [5] reported an AKI incidence of 39.8%; in this study AKI was classified using the KDIGO definition modified to include only serum creatinine. A similar definition was adopted in a prospective observational study of 229 VLBW infants by Koralkar et al. [1] with an AKI incidence of 18%. Stojanović et al. [6] observed an AKI incidence of 26% in a retrospective cohort of 150 preterm neonates, the majority of whom (94.8%) had a birthweight less than 1500 g; they defined AKI as an increase of serum creatinine levels ≥ 26.5 µmol/L compared with basal values. The variations in the reported incidences of neonatal AKI between studies are partly attributable to the differences in patient selection and the nonuniform AKI definitions used. The observed low incidence of KDIGO-defined AKI in our study may, at least in part, reflect the infrequent and non-systematic measurement of plasma creatinine in the study period, a limitation inherent to the retrospective nature of the study.

When examining the demographic data, we divided the study cohort into three groups according to kidney function: AKI group, infants with abnormal P-Crea not fulfilling the AKI definition and normal kidney function. There were no significant differences between the study groups regarding gestational age, birthweight, gender, SGA status, duration of mechanical ventilation, or the incidence of sepsis during the first postnatal week or the incidences of BPD, PDA, NEC, and IVH. Neonatal mortality was significantly higher in the AKI group compared with the other groups, reaching 17% by the end of the first postnatal month. Consistent with our findings, neonatal AKI has been associated with increased mortality in several studies [1, 2, 6, 25]. In our study, infants in the AKI group had the lowest MAP nadir during the first day of life, had a higher intake of volume expanders and received inotropes more frequently than infants in the other groups. Due to these differences in hemodynamics, we adjusted the regression models of fluid and sodium intake and AKI with the average MAP during the first 12 h of life and found that the higher intakes remained significantly associated with an increased risk of AKI. Consistent with our findings, Viswanathan et al. [27] reported a 12.5% incidence of AKI in a case–control study of 472 extremely-low-birthweight (ELBW) infants, identifying low MAP as one of the associated risk factors. Lee et al. [28] demonstrated in a retrospective cohort of 276 ELBW infants an AKI incidence of 56%, with lower GA and inotropic agent utilization independently associated with an increased risk of AKI. Furthermore, Lee et al. [28] identified maternal pre-eclampsia as a protective factor against neonatal AKI, a trend that was also observed in our cohort, although it did not reach statistical significance.

We hypothesized that low fluid intake would be associated with a higher incidence of AKI but failed to demonstrate this in our data. The European Society for Paediatric Gastroenterology, Hepatology and Nutrition (ESPGHAN) guideline recommends a total fluid intake of 80–100 mL/kg/day during the first day of life in ELBW infants weighing less than 1000 g and a 70–90 mL/kg/day intake in those weighing 1000–1500 g [29]. Compared with these recommendations, the total fluid intake in all study groups was high, the lowest median intake being 122 mL/kg/day in the normal kidney function group. The normal kidney function group demonstrated an average physiological weight loss of − 1.2% on day 2, whereas infants in the AKI group had a weight gain of + 2.8% on the same day. According to the ESPGHAN guideline, a 7 to 10% physiological weight loss is considered to be adequate in VLBW infants [29]. Given the overall high fluid intake and modest or absent postnatal weight losses, it can be concluded that volume depletion-related AKI was unlikely to be a predominant etiology of kidney injury in our cohort. Furthermore, the high average fluid intake and minimal postnatal weight loss in the cohort may have resulted in dilutional effects on plasma creatinine concentrations, thereby potentially resulting in a lower number of AKI diagnoses.

In our cohort, AKI was diagnosed on average on the fifth postnatal day—a timing consistent with that reported by Stojanović et al. [6]. This, together with the frequent use of nephrotoxic medications during the early postnatal period in VLBW infants, underscores the importance of systematic monitoring of serum creatinine values, particularly toward the end of the first week of life.

Several studies have shown that a decreasing GA is associated with an increased risk of AKI [3, 6, 30, 31]. As previously discussed, the AWAKEN study reported a U-shaped association between GA and AKI risk, with the lowest incidence observed in infants born between 29 and less than 36 weeks of gestation [2]. In our data, the gestational ages in the AKI group and normal kidney function group were very similar, and no statistically significant differences were observed.

The detailed recordings of fluid management and hemodynamic parameters in a large cohort of VLBW infants represent a major strength of our study. The retrospective design introduces certain limitations. A considerable number of infants were excluded from the analyses due to having fewer than two serum creatinine measurements during the first two postnatal weeks. Among those included in the analyses, serum creatinine was measured three times on average. The relatively infrequent assessment of kidney function during the study period of years 2005–2013 may have led to an underestimation of AKI incidence in our cohort. Following the publication of the AWAKEN study in 2017, our unit implemented a standardized protocol for plasma creatinine monitoring in preterm infants, recognizing the importance of early AKI detection to guide fluid and medication management. Collecting urine output at 24-h intervals also presents a methodological limitation, as the diuresis criterion for AKI might have been met in shorter time frames, such as 6- or 12-h periods. When evaluated over a full 24-h period, shorter episodes of oliguria may have been masked, thus leading to a lower incidence of AKI. Due to missing clinical data, we were unable to calculate the CRIB II score or another validated morbidity index, and thus could not include a standardized measure of overall illness severity in our analysis. Data on urinary sodium excretion were also unavailable.

## Conclusion

High early fluid and sodium intake are associated with an increased risk of AKI in VLBW infants. Early fluid and sodium intake derive largely from infusion fluids used for volume replacement. While ensuring adequate volume resuscitation, it is important to acknowledge that excessive fluid and sodium administration is associated with an increased risk of AKI.

## Supplementary Information

Below is the link to the electronic supplementary material.

High Resolution Image (3.49 MB)

ESM2 (PNG 786 KB)

ESM 1 (PDF 2.12 MB)

## Data Availability

Data is available upon request due to privacy and ethical restrictions.

## Abbreviations

AKI: Acute kidney injury
AWAKEN: Assessment of Worldwide Acute Kidney injury Epidemiology in Neonates
BPD: Bronchopulmonary dysplasia
CI: Confidence interval
ELBW: Extremely-low-birthweight
ESPGHAN: European Society for Paediatric Gastroenterology, Hepatology and Nutrition
GA: Gestational age
IVH: Intraventricular hemorrhage
IQR: Interquartile range
KDIGO: Kidney Disease: Improving Global Outcomes
MAP: Mean arterial pressure
NEC: Necrotizing enterocolitis
NICU: Neonatal intensive care unit
NSAID: Non-steroidal anti-inflammatory agent
OR: Odds ratio
P-CREA: Plasma creatinine
PDA: Patent ductus arteriosus
SD: Standard deviation
SGA: Small for gestational age
THL: The Finnish Institute of Health and Welfare
VLBW: Very low birthweight
