# Supplementary figures and images for: High early fluid and sodium intake as risk factors for acute kidney injury in very-low-birthweight infants
Source: Pediatr Nephrol. 2025 Nov 20;41(4):1191–201. doi: 10.1007/s00467-025-07049-w (PMC12953291; doi:10.1007/s00467-025-07049-w)

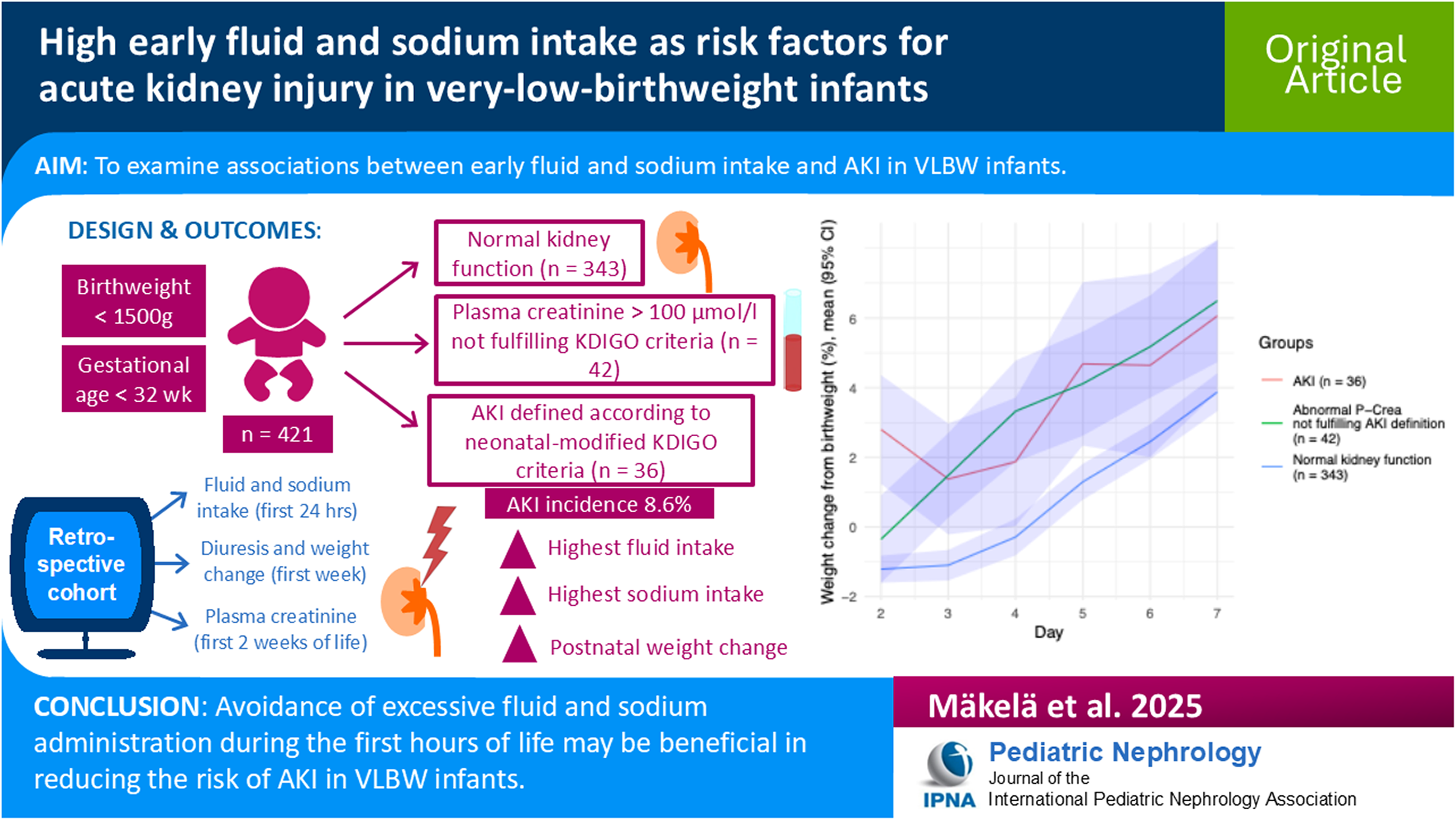

Supplement: Supplementary file 1 — High Resolution Image (3.49 MB) [file 467_2025_7049_MOESM1_ESM.tif]

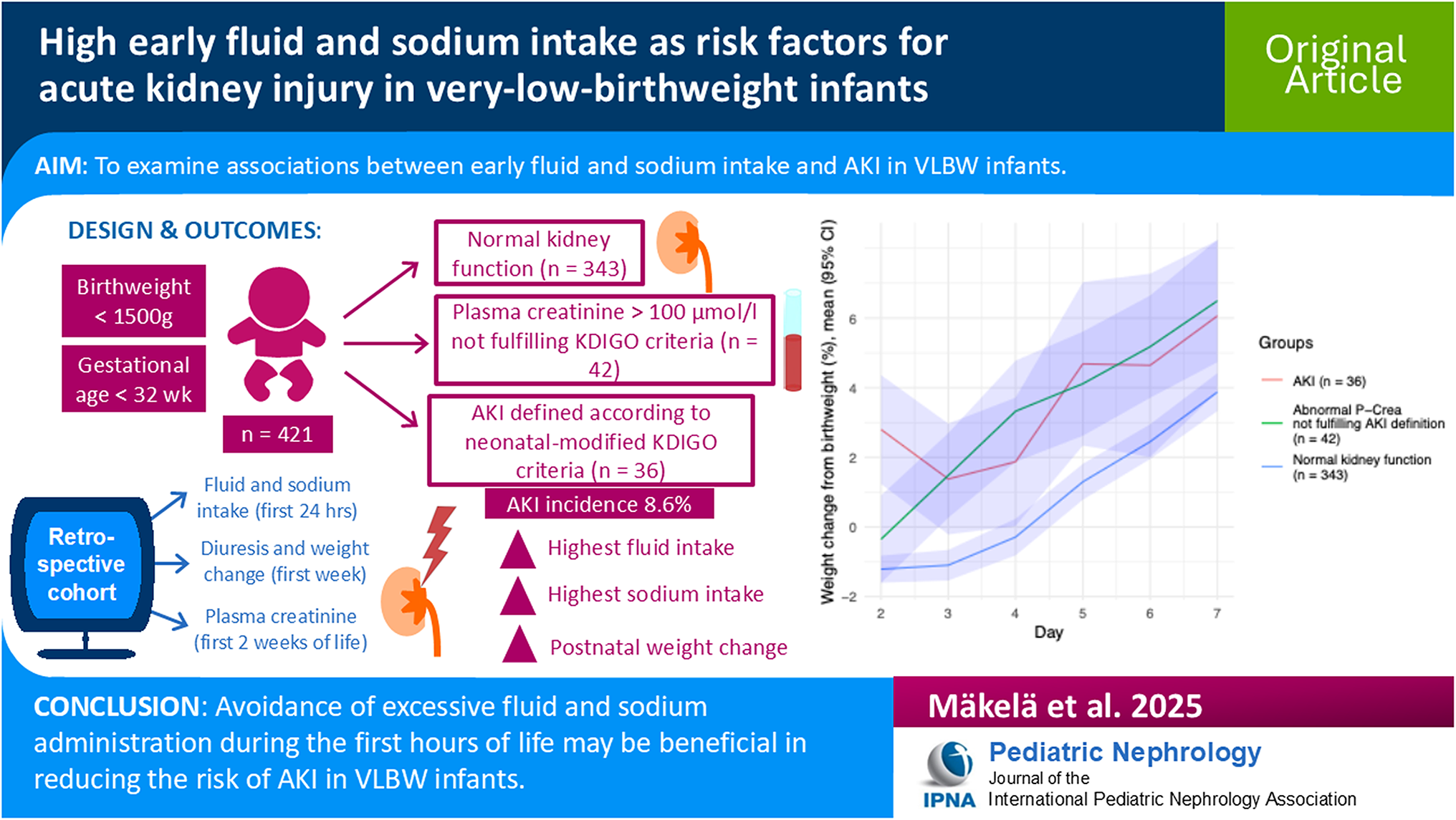

Supplement: Supplementary file 2 — (PNG 786 KB) [file 467_2025_7049_Fig4_ESM.png]
