# Supplementary material for: High early fluid and sodium intake as risk factors for acute kidney injury in very-low-birthweight infants
Source: Pediatr Nephrol. 2025 Nov 20;41(4):1191–201. doi: 10.1007/s00467-025-07049-w (PMC12953291; doi:10.1007/s00467-025-07049-w)
Supplement: Supplementary file 3 — (PDF 2.12 MB) [file 467_2025_7049_MOESM2_ESM.pdf]

Supplementary information for “High early fluid and sodium intake as risk factors for acute kidney injury in very-low-birthweight infants”:

Journal:

Pediatric Nephrology

Authors:

Pauliina M. Mäkelä, Lotta Immeli, Markus Leskinen, Reijo Sund, Timo Jahnukainen, Sture Andersson and Päivi Luukkainen

Corresponding author:

Pauliina M. Mäkelä, New Children’s Hospital, Department of Pediatric Nephrology and Transplantation, Helsinki University Hospital, Helsinki, Finland. Email pauliina.makela@hus.fi.

Supplementary Figure 1. Scatterplot presenting the relationship between sodium intake during the first 24 h of life and the first plasma sodium concentration on postnatal day 1.

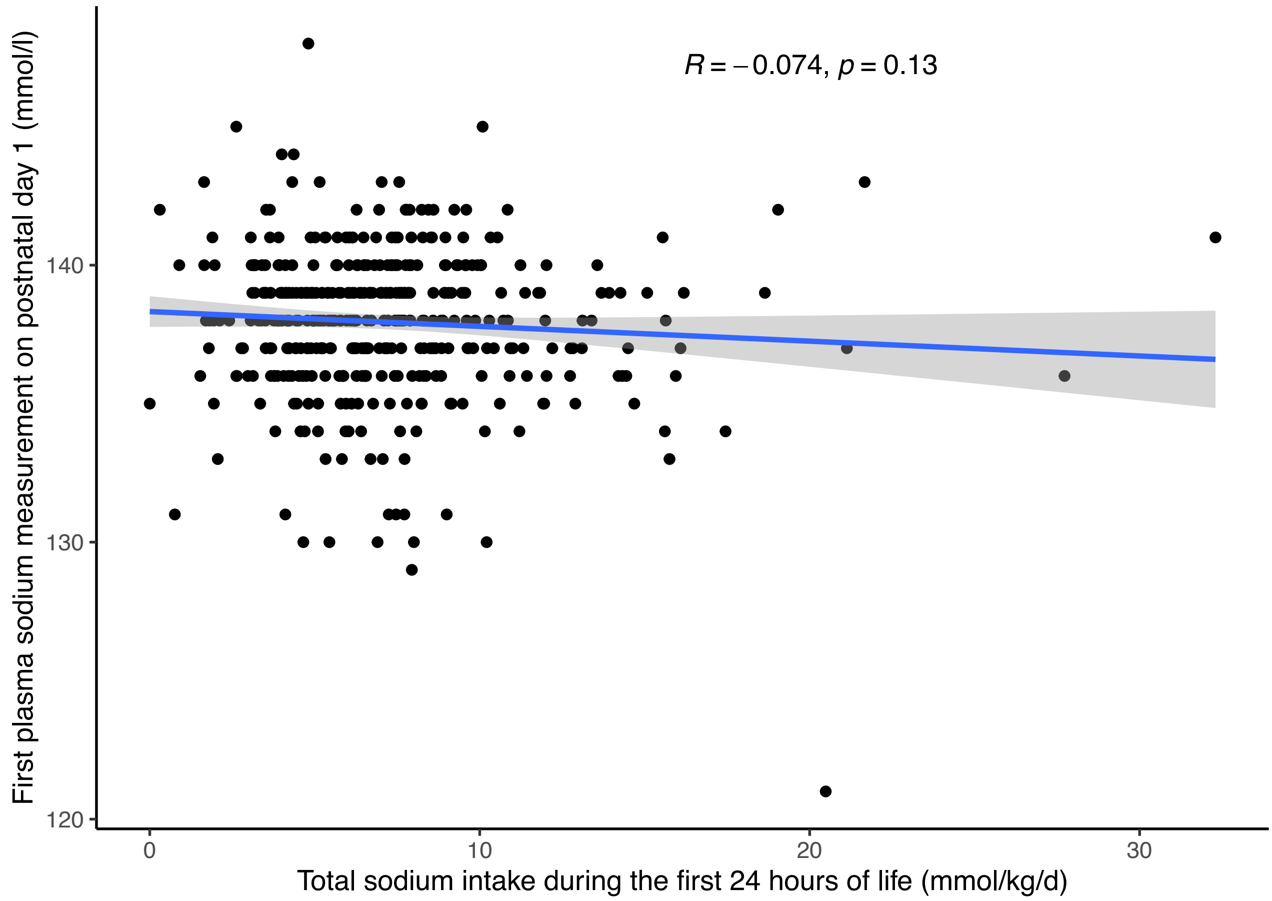

Supplementary Figure 2. Scatterplot presenting the relationship between sodium intake during the first 24 h of life and the first plasma sodium concentration on postnatal day 2.

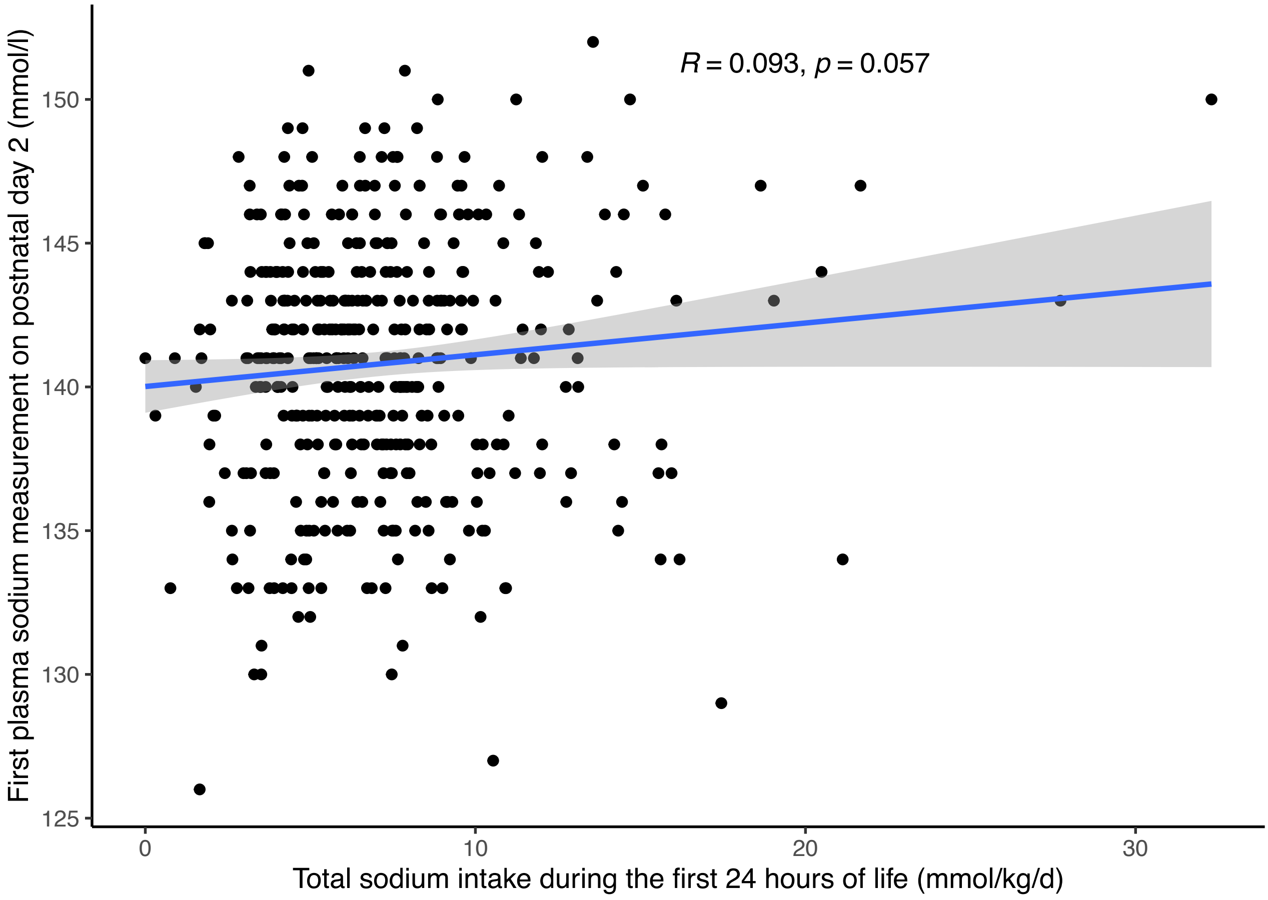

Supplementary Table 1. Plasma sodium concentration during the first 48 hours of life.

	AKI (n=36)	Abnormal P-Crea not fulfilling AKI definition (n=42)	Normal kidney function (n=343)	p value
First plasma sodium concentration on postnatal day 1 (mmol/L), median(min-max)	137.0 (130.0-142.0)	138.0 (130.0-145.0)	138.0 (121.0-148.0)	0.04 (Kruskal-Wallis test)*
Average plasma sodium concentration on postnatal day 1 (mmol/L), median (min-max)	137.7 (131.6-143.0)	137.4 (129.3-146.1)	138.8 (130.3-147.9)	0.04 (Kruskal-Wallis test)*
First plasma sodium concentration on postnatal day 2 (mmol/L), median(min-max)	139.5 (129.0-147.0)	140.0 (127.0-150.0)	141.0 (126.0-152.0)	0.06 (Kruskal-Wallis test)*
Average plasma sodium concentration on postnatal day 2 (mmol/L), median (min-max)	141.2(131.4-148.9)	141.2 (131.6-152.1)	142.0 (131.0-157.1)	0.06 (Kruskal-Wallis test)*

* No significant differences between the groups in pairwise comparison.

Supplementary Table 2. Fluid and sodium intake during the first postnatal week in infants with complete data (n=359), and multivariable logistic regression models predicting the odds of acute kidney injury (AKI).

**A. Cumulative intake during the first postnatal week: median (IQR)**	AKI (n=27)	Abnormal P-Crea not fulfilling AKI definition (n=36)	Normal kidney function (n=296)	p value (Kruskal-Wallis test)
Total fluid intake (mL/kg/wk)	1055 (948-1191)	1113 (978-1226)	1051 (979-1179)	0.59*
Total sodium intake (mmol/kg/wk)	44 (35-60)	45 (35-59)	39 (30-48)	0.018*
* In pairwise comparison, no statistically significant differences between the groups.				
**B. Multivariable logistic regression model predicting the odds of AKI**		95% Confidence interval		
	Odds ratio (OR)	Lower	Upper	p value
Cumulative fluid intake during the first postnatal week (mL/kg/wk)	1.0002	0.997	1.003	0.86
Gestational age (days)	1.017	0.980	1.055	0.37
Small for gestational age (true)	0.849	0.236	2.648	0.79
**C. Multivariable logistic regression model predicting the odds of AKI**		95% Confidence interval		
	Odds ratio (OR)	Lower	Upper	p value
Cumulative sodium intake during the first postnatal week (mmol/kg/wk)	1.034	1.006	1.062	**0.02**
Gestational age (days)	1.030	0.999	1.062	0.06
Small for gestational age (true)	0.527	0.150	1.563	0.28

Supplementary Table 3. Multivariable logistic regression model predicting the odds of acute kidney injury.

		**95% Confidence interval**		
	Odds ratio (OR)	Lower	Upper	p-value
Total fluid intake during the first 24 hours of life (mL/kg/d)	1.015	1.005	1.025	<0.01
Gestational age (days)	1.012	0.985	1.039	0.39
Small for gestational age (true)	0.415	0.125	1.141	0.11
Exposure to NSAIDs during the first 24 hours of life (true)	2.567	0.562	8.606	0.16

Supplementary Table 4. Multivariable logistic regression model predicting the odds of acute kidney injury.

		95% Confidence interval		
	Odds ratio (OR)	Lower	Upper	p-value
Total sodium intake during the first 24 hours of life (mmol/kg/d)	1.13	1.04	1.22	<0.01
Gestational age (days)	1.01	0.98	1.03	0.63
Small for gestational age (true)	0.44	0.13	1.23	0.15
Exposure to NSAIDs during the first 24 hours of life (true)	2.73	0.60	9.14	0.14
